# Multi-Scale Sampling to Evaluate Assemblage Dynamics in an Oceanic Marine Reserve

**DOI:** 10.1371/journal.pone.0033131

**Published:** 2012-03-20

**Authors:** Andrew R. Thompson, William Watson, Sam McClatchie, Edward D. Weber

**Affiliations:** Fisheries Resources Division, Southwest Fisheries Science Center, National Marine Fisheries Service, National Oceanic and Atmospheric Administration (NOAA), La Jolla, California, United States of America; National Institute of Water & Atmospheric Research, New Zealand

## Abstract

To resolve the capacity of Marine Protected Areas (MPA) to enhance fish productivity it is first necessary to understand how environmental conditions affect the distribution and abundance of fishes independent of potential reserve effects. Baseline fish production was examined from 2002–2004 through ichthyoplankton sampling in a large (10,878 km^2^) Southern Californian oceanic marine reserve, the Cowcod Conservation Area (CCA) that was established in 2001, and the Southern California Bight as a whole (238,000 km^2^ CalCOFI sampling domain). The CCA assemblage changed through time as the importance of oceanic-pelagic species decreased between 2002 (La Niña) and 2003 (El Niño) and then increased in 2004 (El Niño), while oceanic species and rockfishes displayed the opposite pattern. By contrast, the CalCOFI assemblage was relatively stable through time. Depth, temperature, and zooplankton explained more of the variability in assemblage structure at the CalCOFI scale than they did at the CCA scale. CalCOFI sampling revealed that oceanic species impinged upon the CCA between 2002 and 2003 in association with warmer offshore waters, thus explaining the increased influence of these species in the CCA during the El Nino years. Multi-scale, spatially explicit sampling and analysis was necessary to interpret assemblage dynamics in the CCA and likely will be needed to evaluate other focal oceanic marine reserves throughout the world.

## Introduction

Marine protected areas (MPAs) potentially influence the dynamics of multiple species because protection from anthropogenic activity is extended, at least to some degree, to all species residing within a geographically bound region. A first step towards understanding the role of MPAs in an ecosystem context is to characterize baseline species assemblages that utilize a reserve and to evaluate how assemblages of species change through time.

An overarching objective of most MPA-based management plans is to augment regional fisheries productivity [1]. Given the diversity of habitats utilized by fishes within and around many MPAs, collecting quantitative, replicable data on multiple species is a major challenge for evaluating the dynamics of species assemblages. This constraint can be overcome to a large extent by collecting fishery independent data such as abundance of early life stages of fishes (i.e., ichthyoplankton). Because ichthyoplankton reflect fish spawning stock biomass [2], [3] this method of sampling has the potential to assess directly whether MPAs impact fishery production from local to regional scales. Determining whether MPAs impact larval output can be complicated, however, because fluctuating environmental conditions are known to induce variability in fish spawning activity independent of reserve effects. To disentangle the impact of underlying environmental dynamics from the influence of MPAs on reproductive output it is necessary to first elucidate how environmental variability affects ichthyoplankton.

Oceanic MPAs are by definition nested within an open, advective system. Although the extent of MPA boundaries are static the spatial extent of water masses can change dramatically among years [4], [5] introducing distinct assemblages of pelagic fishes [6] and zooplankton [7]. It is therefore likely that large-scale oceanographic processes will influence local assemblages within an oceanic MPA in a way that is not reflected solely at the local scale. Thus, a potentially fruitful approach for monitoring assemblage dynamics in an oceanic MPA is to couple detailed, focused sampling within the MPA with broader sampling to monitor the larger region encompassing the MPA.

**Figure 1:**
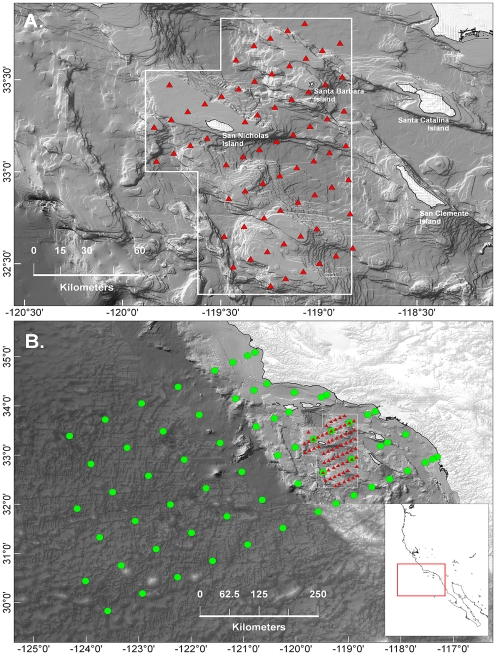
Maps showing the multi-scale sampling domains and locations where samples were collected. (A) Cowcod Conservation Area (CCA) sampling domain. Triangles depict sample sites, and the white line delineates the border of the western CCA. (B) CalCOFI sampling domain. Circles indicate the location of CalCOFI sample sites, triangles the location of CCA sample sites, and white lines the borders of the western and eastern CCAs. The red rectangle in the inset figure delineates the geographic boundary of the area in (B) relative to a broader view of western North America.

The Cowcod Conservation Area (CCA; Fig. 1A) is the largest MPA in Southern California and the only one that includes extensive oceanic and nearshore habitats [8]. The CCA is embedded within the California Cooperative Oceanic Fisheries Investigations (CalCOFI) sampling domain where ichthyoplankton have been sampled regularly since 1951 at sixty-six stations including five within the CCA (Fig. 1B). Although analysis of CalCOFI data provided insight on ichthyoplankton dynamics at the scale of the SCB over decadal time periods (e.g., [9], [10]) understanding of spatial and temporal patterns at finer scales within the CalCOFI domain is limited (but see [11], [12]). To characterize baseline ichthyoplankton assemblages within the CCA, fine-scale surveys were conducted in the winters of the three years (2002–2004) following establishment.

Here, we analyze the CCA data and accompanying CalCOFI data to determine how assemblage dynamics at the larger scale compared with the smaller CCA scale and discern how large scale dynamics affected the fish assemblage at the smaller scale. This study occurred during a transition from La Niña (2002) to El Niño (2003, 2004) conditions [13] which enabled us to assess how fluctuating ocean conditions affected within- and between-year assemblage structure at both spatial scales.

## Methods

### Ethics Statement

Collections were made pursuant to a Memorandum of Understanding (MOU) by and between the California Department of Fish and Game, National Oceanic and Atmospheric Administration (NOAA)/National Marine Fisheries Service (NMFS)/Southwest Region, and NOAA/NMFS/Southwest Fisheries Science Center dated 11 February 1986 in a manner that ameliorated suffering of specimens.

### Study area and sample design

The CCA is a restricted-fishing zone that was established in 2001 in response to population declines of the cowcod rockfish (*Sebastes levis*) [8]. It consists of an approximately 10,878 km^2^ western (Figs. 1A, B) and 260 km^2^ eastern area (Fig. 1B). Our analysis focused on the spatially-continuous western CCA (henceforth CCA; Fig. 1A). Within the CCA it is illegal to fish in waters deeper than 36 m. Although the CCA is entirely on the continental shelf, bottom depths are quite heterogeneous, ranging from sea level at the shores of San Nicholas and Santa Barbara Islands to near 2000 m in multiple basins.

Ichthyoplankton and oceanographic data were collected at two spatial scales: the focal CCA (Fig. 1A), and the larger, approximately 238,000 km^2^, California Cooperative Oceanic Fisheries Investigations (CalCOFI; see [14] for a description of the CalCOFI program) area that encompasses the CCA (Fig. 1B). At both scales sample sites were arranged in a grid with longitudinally separated lines running roughly perpendicular to shore. Sixty-six locations were sampled within the CCA each year with adjacent sample sites separated by approximately 9.5 and 18 km in longitudinal and latitudinal directions, respectively. Measurements were also taken from sixty-six locations at the CalCOFI scale. These sites constitute the “core” CalCOFI stations that have been sampled regularly since 1951 [15]. Adjacent CalCOFI lines were 74-km apart. Within lines, stations were unequally spaced as adjacent sample locations on the continental shelf were closer to one another (10 to 37 km) than seaward stations (74 km) (Fig. 1B).

Icthyoplankton samples and environmental measurements were collected annually in the winters of 2002, 2003, and 2004, during the peak rockfish (*Sebastes* spp.) reproductive period [16]. In 2002 samples at both scales were taken between 24 January and 11 February and in 2003 between 30 January and 15 February. In 2004 the CalCOFI sites were sampled prior (January 5–20) to the CCA sites (February 10–16). Data were collected continuously day and night.

The standard CalCOFI bongo net (71-cm diameter openings, 0.505 mm mesh nets, detachable 0.333 mm mesh cod ends; [11]) protocol was used to sample ichthyoplankton at all site [17]. A flowmeter attached to the net measured volume of filtered water. Samples were preserved in 5% formalin buffered with sodium borate, and all fish larvae were identified to the lowest possible taxonomic level in the laboratory. Most taxa were identified to species (Table S1) but, with the exception of *Sebastes jordani*, *S. paucispinis*, and *S. levis*, rockfish larvae are morphologically indistinguishable and were identified to genus (*Sebastes* spp.). The number of fish larvae under 10 m^2^ surface area (to the maximum depth of a haul) was calculated for each taxon following the standard CalCOFI methodology [18].

Four environmental covariates were sampled at each site. Sea surface temperature (SST) and bottom depth (depth) were taken directly from ship instruments at the initiation of each haul. Small macrozooplankton displacement volume (henceforth “zooplankton”) was calculated for each sample (ml of plankton displacement per 1000 m^3^ of filtered water) [19]. We also estimated chlorophyll *a* values based on satellite imagery for the 30-day period encompassing each survey. This variable, however, was consistently highly correlated with at least one of the other covariates, and we therefore excluded chlorophyll *a* from further analysis.

### Environmental Variability

We expected that the ichthyoplankton assemblage would be affected by oceanographic conditions that changed between the 2002 La Niña and 2003–2004 El Niño [13]. We determined if SST and zooplankton within our sample frames reflected oceanographic conditions expected during La Niña (cool SST, high zooplankton) and El Niño (high SST, low zooplankton) years in the Southern California region [15] and whether the sampling perspective affected the patterns.

### Change in assemblage structure

To help visualize assemblage dynamics among years we first performed separate principle components analyses (PCA) on the site by species matrices from the CCA and CalCOFI scales and plotted site scores from the first two PC axes (PC1 and PC2). We then used redundancy analysis (RDA) to determine if there were differences in assemblage structure between years at the CCA and CalCOFI sample domains. Sample year was treated as a discrete variable and overall significance was assessed at α = 0.05 based on 1000 permutations of the data. If an overall difference was detected, comparisons between pairs of continuous years were made with significance levels adjusted to account for multiple comparisons (α = 0.017). Adjusted *R* ^2^ [20] values were also reported to quantify the unbiased coefficient of determination for each test. Prior to this and all other analyses on assemblages (see below) taxa were removed that were not found in at least 5% of the samples and stations were taken out that contained less than three individual larvae. One station was removed from the 2003 CalCOFI data set and nine from the 2004 CalCOFI analysis due to the low (0 or 1) number of captured larvae. Fish abundances were Hellinger-transformed prior to ordination because this transformation has been shown to produce unbiased results in Euclidian-based multivariate analysis such as PCA and RDA when zero species counts are prevalent [21].

### Effect of covariates on interannual dynamics

We discerned the individual and combined effects of SST, zooplankton, depth, and year on CCA and CalCOFI assemblage dynamics. First, to evaluate the effects of the covariates on an entire assemblage, we conducted variance partitioning of scale-specific RDAs that modeled the covariates SST, zooplankton, depth and time against assemblage structure [20]. We included time as a distinct, categorical covariate to ascertain how much variance was explained by year of sampling that was not attributable to the sampled environmental parameters. Second, to better elucidate the dynamics of particular groups of species, we extracted the first two PC eigenvectors from the unconstrained, scale-specific PCAs that were based on the site by species matrices. We then used linear models to calculate how much of the variation in PC1 and PC2 was explained by the covariates. Finally, we used variance partitioning to ascertain the unique and shared contribution of each covariate to the explained variation of PC 1 and PC2 at each scale.

### Effect of covariates on within-year distributions

We analyzed the horizontal distribution of assemblages within each year at both scales. We again evaluated how well covariates explained year-specific assemblage structure first using RDA and second linear models of PC1 and PC2 from PCAs of the site by species matrix from each assemblage at both scales. We then used variance partitioning to determine the amount of variation that was explained by each covariate. In addition to the environmental covariates (depth, SST and zooplankton) we included in these analyses spatial covariates. Spatial covariates provide insight towards processes affecting species distribution because they identify nonrandom distribution patterns that are not fully explained by the measured environmental covariates [21]. Such spatial effects indicate that unmeasured endogenous (i.e., behavioral or ecological) or exogenous (i.e., environmental parameters) processes affect an assemblage, and/or there is a mismatch between the scale of sampling and the response of species to environmental variables [22]–[24]. Thus, spatial covariates provide valuable clues regarding processes that affect assemblages that are not identified by the sampled environmental variables [24].

Spatial covariates were generated specifically for the CCA and CalCOFI domains using the principle coordinates of neighbor matrices (PCNM) method. PCNM produces eigenvectors based on a connectivity matrix (minimum spanning tree) of the geographical coordinates of sample sites (see Figure 1 in [25] for an illustration that describes the derivation of PCNM eigenvectors). The resultant spatial covariates (PCNM variables) depict spatial relationships among sample sites at a continuum of spatial scales within the bounds of the sample area [21]. PCNM variables with large eigenvalues describe broad-scale spatial relationships and vice versa.

We utilized a conservative approach for including PCNM variables in the analyses to avoid overfitting [26]. We reduced the number of PCNM variables by using a conservative forward selection technique based on adjusted *R* ^2^ values and a cutoff of *p* = 0.05 [27] and limited to five the number of PCNM variables in any one analysis [28].

PCA, RDA, variance partitioning, and forward selection analyses were carried out using the statistical software R version 2.11.0 with the packages Vegan 1.17–2 [29] and packfor [27]. We generated PCNM eigenvectors using SAM v4.0 [30]. Maps were produced using ArcGIS version 10.0.

## Results

### Environmental dynamics

**Figure 2:**
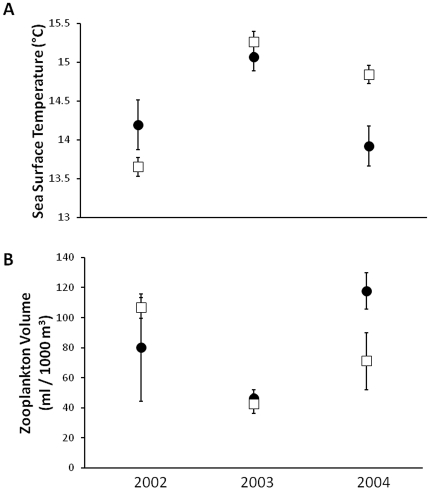
Interannual environmental variation at both scales. (A) Mean (±2SE) sea surface temperature; (B) Zooplankton volume. The filled circles are from the CCA and the open squares from the CalCOFI sample domains.

Interannual patterns of SST and zooplankton conformed to previous findings of low SST and high zooplankton during a La Niña and vice versa during an El Niño [15] at the large CalCOFI scale in each year, and at the smaller CCA scale in 2002 and 2003 but not 2004 (Fig. 2). In the CCA SST increased by an average (±2 SE) of 0.88±0.16°C between 2002 and 2003 and then decreased by 1.15±0.14°C between 2003 and 2004 such that the SST was lowest among the sample years during the 2004 El Niño (Fig. 2A). At the CalCOFI scale SST also increased from 2002 to 2003 (1.56±0.21°C) but decreased only slightly (0.44±0.22°C) from 2003 to 2004 with the lowest average temperature occurring during the 2002 La Niña (Fig. 2A). Zooplankton consistently displayed an inverse pattern to SST at both scales (Fig. 2B).

### Change in assemblage structure

**Figure 3:**
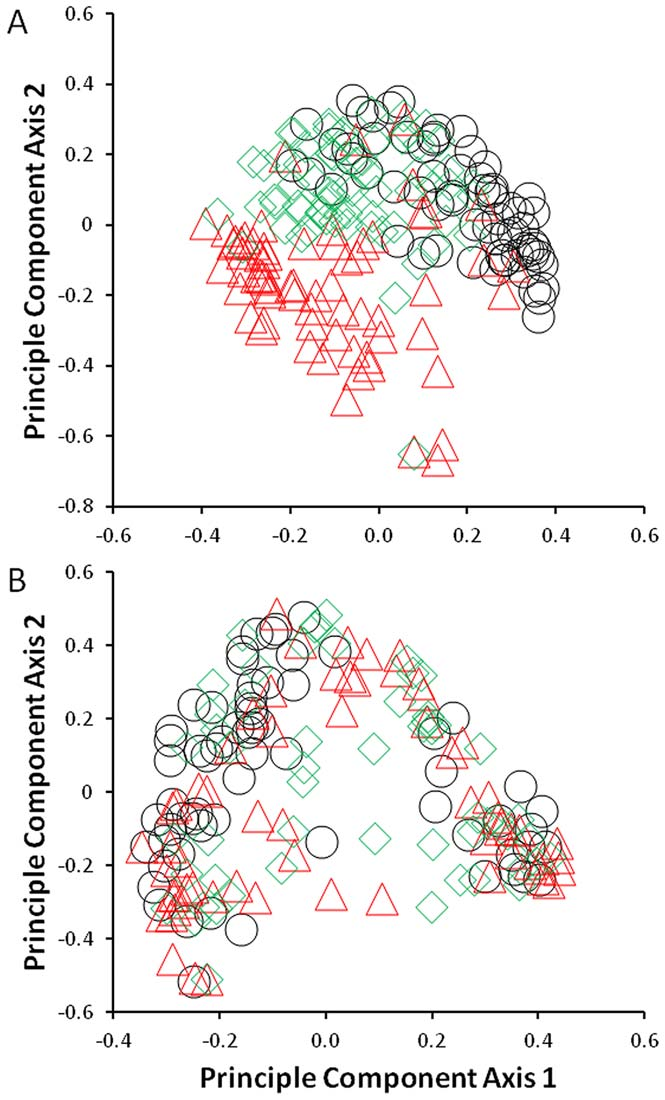
Variability in assemblage structure among years at both scales. Scores of PC1 and PC2 of each station from 2002 (circles), 2003 (triangles), and 2004 (diamonds) are shown for the (A) CCA and (B) CalCofi scales. See Table 2 for taxa loadings on each axis.

The larval assemblage was much more dynamic at the smaller CCA than the larger CalCOFI scale over the three year period (Fig. 3). Within the CCA the overall RDA model of assemblage structure versus sample year was highly significant (*R* ^2^ = 0.23, p<0.001) as were pairwise comparisons between individual years (2002 vs 2003 *R* ^2^ = 0.22, 2003 vs. 2004 *R* ^2^ = 0.12; p<0.001 for both). Differences between 2002 and 2003 in the CCA reflected an increase in the influence of benthic rockfishes (*Sebastes* spp., *S. jordani*, and *S. paucispinis*) and oceanic species (*N. ritteri*, *P. crockeri*, *S. californiensis*, *D. atlanticus*) relative to coastal-oceanic species (*E. mordax*, *L. stilbius*, *M. productus*) (Table 1; Figs. S1, S2, S3, S4, 3A, 4; see [31] for the definition of species-specific habitat associations). From 2003 to 2004 the influence of the oceanic species declined while that of the coastal-oceanic species increased (Table 1; Figs. S1, S2, S3, S4, 3A, 4). Although the overall model at the CalCOFI scale was significant (p<0.01) the coefficient of determination (*R* ^2^ = 0.02) was much lower than in the CCA. Pairwise comparisons of CalCOFI years indicated that while the assemblage differed between 2002 and 2003 (adj *R* ^2^ = 0.06; p<0.01) it did not change significantly from 2003 to 2004 (adj *R* ^2^ = 0.01; p>0.05). The difference between 2002 and 2003 at the CalCOFI scale was driven primarily by an increase in the influence of oceanic species (*N. ritteri*, *P. crockeri*, *S. californiensis*, *D. atlanticus*) relative to *M. productus* and *Sebastes spp.* in 2003 (Table 1; Figs. S5, S6, S7, S8, 3B, 5).

**Table 1:** Frequency of occurrence, mean abundance, and proportion of abundance constituted by individual ichthyoplankton taxa for each sample year within the CCA and CalCOFI sampling domains.

			CCA Domain						CalCOFI Domain					
			Frequency of Occurrence			Mean Abundance			Frequency of Occurrence			Mean Abundance		
Taxon	scientific name	common name	2002	2003	2004	2002	2003	2004	2002	2003	2004	2002	2003	2004
Clupeidae														
	*Sardinops sagax*	Pacific sardine	0.09	0.01	0.13	32.85	0.07	0.81	0	0.03	0	0	0.36	0
Engraulidae														
	*Engraulis mordax*	Northern anchovy	0.91	0.31	0.63	302.72	24.26	15.60	0.35	0.29	0.08	12.80	18.49	0.51
Argentinidae														
	*Argentina sialis*	Pacific argentine	0.20	0.01	0.09	1.04	0.08	0.51	0.03	0.02	0.05	0.22	0.07	0.29
Bathylagidae														
	*Leuroglossus stilbius*	Cal. smoothtongue	1.00	0.79	0.91	286.04	29.01	47.84	0.63	0.28	0.30	52.74	4.29	13.80
	*Lipolagus ochotensis*	Popeye blacksmelt	0.92	0.81	0.94	44.32	11.81	31.37	0.66	0.37	0.31	30.85	3.24	8.59
Gonostomatidae														
	*Cyclothone signata*	Showy bristlemouth	0	0.09	0.09	0	0.72	0.65	0.08	0.15	0.17	0.68	1.67	1.14
Sternoptychidae														
	*Argyropelecus sladeni*	Lowcrest hatchetfish	0.18	0.04	0.04	1.02	0.22	0.21	0.12	0.08	0.06	0.98	0.42	0.44
	*Danaphos oculatus*	Bottlelight	0.09	0.07	0.13	0.45	0.46	0.73	0.12	0.09	0.11	1.24	0.42	0.77
Phosichthyidae														
	*Vinciguerria lucetia*	Panama lightfish	0.02	0.01	0.07	0.07	0.07	0.71	0.18	0.18	0.08	4.61	3.27	0.42
Stomiidae														
	*Stomias atriventer*	Blackbelly dragonfish	0.08	0.04	0.10	0.59	0.24	0.49	0.02	0.06	0.02	0.16	0.28	0.07
Idiacanthidae														
	*Idiacanthus antrostomus*	Pacific blackdragon	0	0.01	0	0	0.08	0	0.12	0.05	0.06	0.76	0.34	0.28
Paralepididae														
	*Lestidiops ringens*	Slender barracudina	0.02	0.16	0.12	0.07	0.95	0.56	0.05	0.14	0.03	0.29	0.68	0.20
Myctophidae														
	*Ceratoscopelus townsendi*	Dogtooth lampfish	0	0	0	0	0	0	0.11	0.09	0.02	1.43	0.49	0.07
	*Nannobrachium ritteri*	Broadfin lampfish	0.27	0.49	0.33	2.04	4.76	3.36	0.15	0.34	0.27	1.53	4.23	2.17
	*Stenobrachius leucopsarus*	Northern lampfish	0.98	0.93	0.99	148.66	53.10	69.53	0.62	0.66	0.50	44.45	24.50	18.76
	*Diogenichthys atlanticus*	Longfin lanternfish	0.08	0.34	0.28	0.45	2.29	1.55	0.25	0.29	0.25	5.76	3.27	2.26
	*Protomyctophum crockeri*	Cal. flashlightfish	0.59	0.46	0.43	7.59	4.47	3.81	0.48	0.49	0.45	7.33	6.72	4.23
	*Symbolophorus californiensis*	Cal. lanternfish	0	0.15	0.13	0	1.13	0.88	0.12	0.25	0.13	1.54	3.20	1.29
	*Tarletonbeania crenularis*	Blue lanternfish	0.42	0.27	0.04	3.49	2.19	0.20	0.20	0.15	0.08	2.49	1.01	0.56
Merlucciidae														
	*Merluccius productus*	Pacific hake	1.00	0.27	0.97	252.80	8.79	117.29	0.62	0.14	0.19	338.59	1.42	9.23
Scorpaenidae														
	*Sebastes aurora*	Aurora rockfish	0.15	0.06	0.10	0.94	0.62	0.73	0.02	0.02	0	0.30	0.07	0
	*Sebastes goodei*	Chillipepper rockfish	0.03	0.09	0.19	0.23	0.61	1.66	0.02	0.03	0	0.16	0.13	0
	*Sebastes jordani*	Shortbelly rockfish	0.82	0.69	0.87	56.48	39.22	27.95	0.28	0.28	0.14	38.35	8.36	3.04
	*Sebastes levis*	Cowcod rockfish	0.11	0.15	0.24	0.88	0.79	2.19	0	0.06	0	0	0.29	0
	*Sebastes paucispinis*	Bocaccio rockfish	0.44	0.64	0.84	6.91	8.37	15.51	0.14	0.17	0.11	1.74	1.81	7.08
	*Sebastes* spp.	?? rockfish	0.88	0.94	0.99	250.07	135.34	345.92	0.55	0.43	0.30	43.59	35.59	32.00
Hexagrammidae														
	*Zaniolepis latipinnis*	Longspine combfish	0.02	0.03	0.13	0.07	0.15	0.84	0.02	0.02	0	0.15	0.07	0
Crangidae														
	*Trachurus symmetricus*	Jack mackerel	0	0.15	0.03	0	1.84	0.23	0.02	0.02	0	0.07	0.36	0
Gobiidae														
	*Rhinogobiops nicholsii*	Blackeye goby	0.30	0.15	0.51	2.14	1.22	3.49	0.02	0.06	0.09	0.15	0.28	0.64
Centrolophidae														
	*Icichthys lockingtoni*	Medusafish	0.03	0	0.03	0.35	0	0.23	0.12	0.02	0	1.25	0.07	0
Paralichthyidae														
	*Citharichthys sordidus*	Pacific sanddab	0.44	0.04	0.66	6.94	0.23	9.81	0.34	0.09	0.20	4.72	0.68	1.94
	*Citharichthys stigmaeus*	Speckled sanddab	0.42	0.15	0.43	3.85	0.75	2.93	0.26	0.06	0.08	5.46	0.58	0.73

Only taxa observed in at least 5% of the stations in at least one of the years are shown; a complete list of sampled taxa is provided in Table S1.

### Effect of covariates on interannual dynamics

The response of the assemblage to environmental fluctuation was much more predictable at the larger than the smaller scale. Variance partitioning of RDA models and linear models of individual PC axes from the PCAs of the original site by species matrices (Table 2) showed that environmental parameters explained much more of the variation in assemblage structure at the CalCOFI than the CCA scale (Table 3). Further, in the CCA the amount of variation explained purely by time (i.e., time with other variables factored out) was greater than that characterized by the environmental covariates for both the RDA and linear model of PC1, which characterized a dichotomy between stations with benthic versus coastal-oceanic taxa (Table 2, 3). At the CCA scale only SST was relatively effective in describing the variability of a linear model of PC2 (coastal-oceanic and benthic versus oceanic taxa; Tables 2, 3). At the CalCOFI scale, by contrast, pure time was unimportant for all analyses, and the environmental covariates were particularly effective in explaining the interannual variability in PC1 (72%), which separated oceanic versus coastal-oceanic and benthic taxa (Tables 2, 3).

**Table 2:** Taxa loadings on the first two principle component axes from a PCA of the site by species matrix of samples from the CCA and CalCOFI sampling domains using data from all years.

Sample	Eigen-	Eigen-	Proportion	Species with	Habitat	Species with	Habitat
Domain	vector	value	variance	Positive loadings	Affinities	Negative loadings	Affinities
CCA	PC1	0.11	0.38	*L. stilbius* (0.78), *E. mordax* (0.61), *M. productus* (0.48)	coastal-oceanic	*Sebastes* spp. (−1.21), *S. jordani* (−0.34), *S. paucispinis* (−0.27)	benthic
	PC2	0.05	0.15	*M. productus* (0.80), *Sebastes* spp. (0.32), *E.mordax* (0.27),	coastal-oceanic, benthic	*N. ritteri* (−0.32), *L. ochotensis* (−0.26), *P. crockeri* (−0.26),	oceanic
				*S. jordani* (0.12)		S. californiensis (−.16), *D. atlanticus* (−0.14)	
CalCOFI	PC1	0.18	0.27	*P. crockeri* (0.77), *D. atlanticus* (0.60), *S. californiensis* (0.42),	oceanic	*Sebastes* spp. (−0.80), *M. productus* (−0.51), *L. stilbius* (−0.40),	coastal-oceanic, benthic
				*V. lucetia (0.42)*, *N. ritteri* (0.36), *C. signata* (0.25)		*S. leucopsarus* (−0.39), *E. mordax* (−0.34), *S. jordani* (−0.32)	
	PC2	0.10	0.15	*Sebastes* spp. (0.70), *S. jordani* (0.23), *S. californiensis* (0.19), *E. mordax* (0.17), *D. atlanticus* (0.17), *V. lucetia* (0.16)	benthic, oceanic, and coastal-oceanic	*S. leucopsarus* (−0.69), *L. ochotensis* (−0.65), *L. stilbius* (−0.24)	oceanic, coastal-oceanic

Habitat affinities as defined by [31] (oceanic: *N. ritteri*, *P. crockeri*, *S. californiensis*, *D. atlanticus*, *S. leucopsarus*; coastal-oceanic: *L. stilbius*, *L. ochotensis*, *M. productus*, *E. mordax*; benthic: *Sebastes* spp., *S. jordani*, *S. paucispinis*) of taxa with positive and negative loadings are shown for each axis. Table 1 providesfull species and common names.

**Table 3:** Adjusted *R* ^2^ values from partitioning of the amount of variation explained by depth (D), sea surface temperature (SST), zooplankton volume (Z) and year of sampling (time) for interannual analysis of assemblage structure at the CCA and CalCOFI scales.

Sample Domain	Analysis	D	SST	Z	Time	D+SST	D+Z	SST+Z	D+Z+SST	Pure Time [T | D, SST, Z]	Total variation explained	Residuals
CCA	RDA	0.05	0.06	0.03	0.23	0.11	0.09	0.06	0.11	0.19	0.30	0.70
	linear model of PC1	0.09	0	0	0.40	0.1	0.1	0.01	0.10	0.42	0.51	0.49
	linear model of PC2	0.08	0.34	0.17	0.34	0.37	0.24	0.35	0.39	0.06	0.45	0.55
CalCOFI	RDA	0.14	0.12	0.04	0.02	0.22	0.16	0.12	0.22	0.01	0.23	0.77
	linear model of PC1	0.51	0.43	0.13	0.03	0.72	0.58	0.44	0.72	0.00	0.72	0.28
	linear model of PC2	0.05	0.06	0	0	0.16	0.06	0.06	0.16	0.01	0.17	0.83

Pure time indicates the fraction of variation explained by time after factoring out the shared variation explained by the environmental covariates. PC axes are extracted from a PCA of the site by species matrix.

### Effect of covariates on within-year distributions

Variance partitioning of models relating environmental and spatial covariates to assemblage structure at each scale within years revealed two main findings. First, the amount of variation explained by the environmental covariates (i.e., depth+SST+zooplankton) measured in the CCA was quite different among years, ranging from 7 to 22% for the RDA models and 14 to 45% for the linear models of PC1 (Table 4). At the CalCOFI scale, by contrast, the amount of variation explained by the environment was consistently higher as RDA models explained between 17 and 30% and linear models of PC1 between 66 and 79% of the variance in assemblage structure (Table 4). Second, the amount of variation explained purely by the spatial covariates (i.e., space with other covariates factored out) in the RDA models was consistently similar to the environmental covariates at the CCA scale. At the CalCOFI scale, however, spatial covariates explained much less of the variation than the environment for both RDA models and linear models of PC1 and PC2 (Table 4).

**Table 4:** Adjusted *R* ^2^ values from partitioning of the amount variation explained by depth (D), sea surface temperature (SST), zooplankton volume (Z) and PCNM eigenvectors (space) for intraannual analysis at the CCA and CalCOFI scales in each sample year.

Sample Domain	Analysis	D	SST	Z	Space	D+SST	D+Z	SST+Z	D+Z+SST	Pure Space [S | D, SST, Z]	Total variation explained	Residuals
CCA 2002	RDA	0.13	0.12	0.01	0.34	0.18	0.15	0.12	0.22	0.21	0.39	0.61
	linear model of PC1	0.31	0.32	0	0.49	0.45	0.34	0.31	0.45	0.16	0.61	0.39
	linear model of PC2	0.05	0	0.00	0.45	0.04	0.04	0	0.02	0.44	0.46	0.53
CCA 2003	RDA	0.02	0.03	0.02	0.17	0.05	0.04	0.05	0.07	0.11	0.17	0.82
	linear model of PC1	0.03	0.06	0.05	0.30	0.10	0.08	0.12	0.14	0.00	0.14	0.86
	linear model of PC2	0.02	0.02	0.05	0.54	0.04	0.08	0.08	0.10	0.44	0.55	0.45
CCA 2004	RDA	0.08	0.02	0.01	0.14	0.10	0.09	0.03	0.10	0.11	0.21	0.79
	linear model of PC1	0.22	0.00	0.02	0.21	0.21	0.22	0.01	0.21	0.21	0.42	0.58
	linear model of PC2	0.09	0.15	0	0.28	0.18	0.08	0.14	0.17	0.14	0.31	0.69
CalCOFI 2002	RDA	0.14	0.16	0.07	0.23	0.24	0.18	0.16	0.24	0.09	0.33	0.67
	linear model of PC1	0.37	0.52	0.24	0.55	0.66	0.53	0.51	0.66	0.11	0.77	0.23
	linear model of PC2	0.13	0.00	0	0.15	0.11	0.11	0.00	0.10	0.17	0.27	0.73
CalCOFI 2003	RDA	0.23	0.10	0.04	0.26	0.30	0.23	0.13	0.30	0.03	0.33	0.67
	linear model of PC1	0.69	0.23	0.12	0.67	0.79	0.69	0.30	0.79	0.06	0.85	0.06
	linear model of PC2	0.02	0.09	0	0.13	0.16	0.00	0.10	0.15	0.05	0.20	0.80
CalCOFI 2004	RDA	0.11	0.15	0.00	0.21	0.18	0.10	0.15	0.17	0.06	0.23	0.77
	linear model of PC1	0.49	0.69	0.01	0.63	0.75	0.50	0.70	0.75	0.04	0.79	0.21
	linear model of PC2	0.01	0.00	0	0.32	0.09	0.00	0.00	0.07	0.24	0.32	0.68

Pure space indicates the fraction of variation explained by the spatial covariates after factoring out the shared variation explained by the environmental covariates. PC axes are extracted from a PCA of the site by species matrix.

## Discussion

The ichthyoplankton assemblage was much more dynamic within the CCA than the CalCOFI area over the three-year survey period that was characterized by a transition from La Niña to El Niño oceanographic conditions. During the La Niña in 2002 the CCA was dominated by species with coastal-oceanic habitat affinities (as described by [31]) such as *E. mordax*, *M. productus*, and *L. stilbius* as well as benthic rockfishes. During the 2003 El Niño, however, there was a decline in the influence of the coastal-oceanic species and an increase in rockfishes and several oceanic species (*N. ritteri*, *D. atlanticus*, *S. californiensis*). In 2004, *E. mordax*, *L. stilbius* and, especially, *M. productus*, increased (although not to 2002 levels). These results suggest that the coastal-oceanic species moved out of the CCA between 2002 and 2003 as the oceanic species moved in, and then returned to the area in 2004. They also highlight how variable the ichthyoplankton assemblage can be in this region at an annual time scale.

Previous ichthyoplankton research in this area characterized similar patterns but averaged over longer time frames. Hsieh et al. [9] examined the distribution and abundance of oceanic (e.g., *N. ritteri*, *P. crockeri*, *L. ochotensis*, *D. atlanticus*) and coastal-neritic (e.g., *E. mordax*, *M. productus*, *S. sagax*) species in association with a regime shift [4], [5] from cool (1950–1976) to warm (1977–1999) conditions. They found that the abundance of oceanic species expanded and encroached shoreward during the warm period whereas the coastal-neritic species retreated shoreward and northward at this time. Similarly, ichthyoplankton abundances of species with subtropical affinities increased and moved shoreward throughout California and Baja California, Mexico, following a transition from cool, La Niña conditions in 1954–56 to warm, El Niño conditions in 1958–1959 [32]. Our results build on these analyses of longer time series by demonstrating how abruptly the ichthyoplankton assemblages can change over a relatively short time frame.

Comparison of the relationship between covariates and assemblage structure at the CCA and CalCOFI scales revealed two main points. First, although the CCA assemblage changed through time, the sampled environmental variables explained relatively little of the variation. At the CalCOFI scale, however, environmental covariates better explained the dynamics of the assemblage. Second, purely temporal (year) and spatial (PCNM) covariates consistently explained more of the variation in assemblage structure than the measured environmental covariates at the CCA scale, but the opposite was true at the CalCOFI scale. These results may be caused by multiple nonexclusive factors [33]. One possibility is that the variation captured by year and unexplained spatial covariates within the CCA was induced by unmeasured environmental factors such as salinity [34], mixed layer depth [35] or dissolved oxygen concentration [10]. Another possibility is that the unexplained time and space variables were proxies for biological interactions. Biological processes such as schooling behavior, competition, and migration/dispersal can induce spatial clustering independent of environmental effects either by concentrating species within a small portion of suitable habitat or by causing species to occupy suboptimal habitat [33]. Because most of the larvae captured in our study were in the very early stages of development and were poor swimmers it is unlikely that behavioral processes had a large effect on horizontal distribution. However, if behavior influenced the distribution of spawning adults then biological interactions could be reflected in larval distributions.

A third possibility is that there was a mismatch between the scale of sampling within the CCA and the response of species to environmental variability. This explanation is supported by recent theoretical work indicating that if species respond to environmental fluctuation at a scale larger or smaller than a sample frame, then there is a tendency for the amount of variation that is explained by spatial covariates to increase relative to environmental parameters [23]. In our case, it is possible that at least the pelagic species responded to environmental variability at a scale that more closely matched the CalCOFI than the CCA sampling domain. For example, the range of SST across the CalCOFI region was 11.5–17.2°C during the study period but across the CCA it was only between 12.7 and 16.2°C. The relatively narrow range of temperatures encountered in the CCA likely had more subtle effects on fish distributions than did the large range of temperatures across the CalCOFI region. In particular, examination of the assemblage from the CalCOFI scale suggests that the assemblage was affected by shifting water mass boundaries as PC1 scores correlated highly with SST. CalCOFI PC1 separated stations characterized by taxa with oceanic affinities (e.g., *N. ritteri*, *P. crockeri*, and *S. californiensis*) from those with coastal-pelagic and benthic habitat association (e.g., *Sebastses* spp., *E. mordax*, *S. leucopsarus* and *M. productus*), and the distribution of these taxa shifted among years. In 2002 stations with high PC1 scores (i.e., oceanic species) clustered in the southwest portion of the sample domain. In addition, stations west of the shelf in the northern part of the CalCOFI domain contained coastal species, suggesting offshelf transport in 2002, a pattern also identified for *S. sagax* in spring of that year [13]. In 2003, by contrast, oceanic species impinged upon the shelf and into the CCA together with warmer water. Despite the large-scale movement of warm water and broad-scale warming in 2003 and 2004 relative to 2002, SST in the CCA was actually lower in 2004 than 2002, perhaps reflecting local upwelling in 2004. This discrepancy between large-scale and local environmental dynamics likely weakened the environment-species relationship in the CCA.

Results from the CalCOFI scale demonstrate that ichthyoplankton assemblage structure is affected by the distribution of large-scale water masses that can have spatially dynamic boundaries [32]. Similar large-scale processes were documented in other parts of the world. For example, ichthyoplankton assemblages in the western Mediterranean were partitioned by two surface water masses, and the distribution of the water masses and assemblages varied seasonally [36]. Similarly, the spatial structure of the ichthyoplankton assemblage throughout the equatorial Pacific varied in response to extended periods of warmth over a 13-year period [37]. In addition, ichthyoplankton assemblages off the coast of Oregon varied significantly in response to warming and cooling of surface waters between 1999 and 2006 [38], [39]. Together, these findings demonstrate that although ichthyoplankton assemblages are often spatially segregated, the locations where particular assemblages are found can vary dramatically through time in response to environmental forcing [40]–[42].

**Figure 4:**
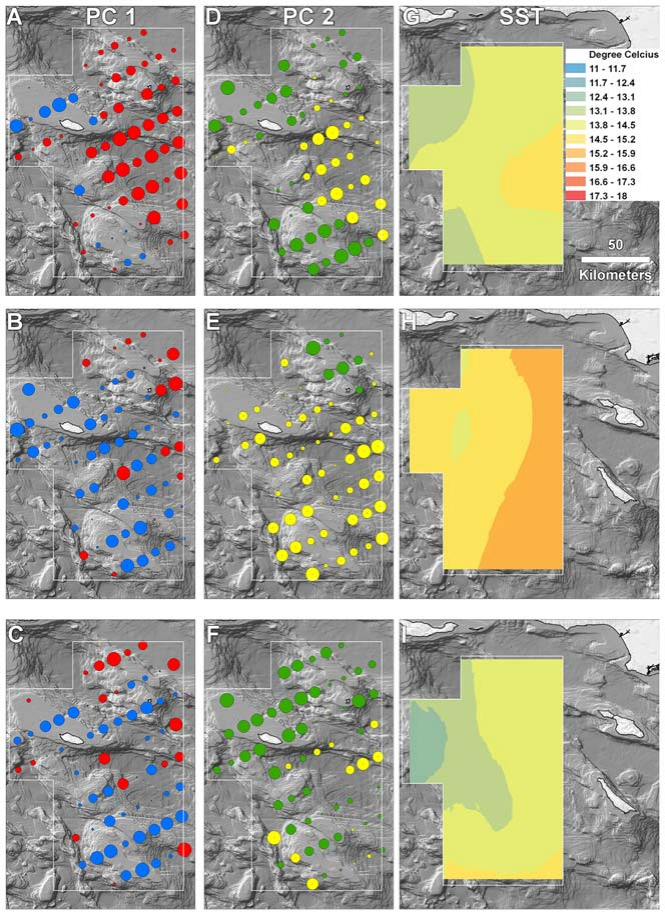
Spatial and temporal variability in assemblage structure and SST at the CCA scale. Principle component 1 (PC1) scores of stations from (A) 2002, (B) 2003 and (C) 2004. Red circles represent positive and blue circles negative loadings on PC1. The size of eachcircle is proportional to PC absolute value. PC2 scores from (D) 2002, (E) 2003 and (F) 2004. Green circles represent positive and yellow circles negative loadings on PC2. See Table 2 for taxa loadings on each axis. Krig-based images of sea surface temperature from (G) 2002, (H) 2003 and (I) 2004.

**Figure 5:**
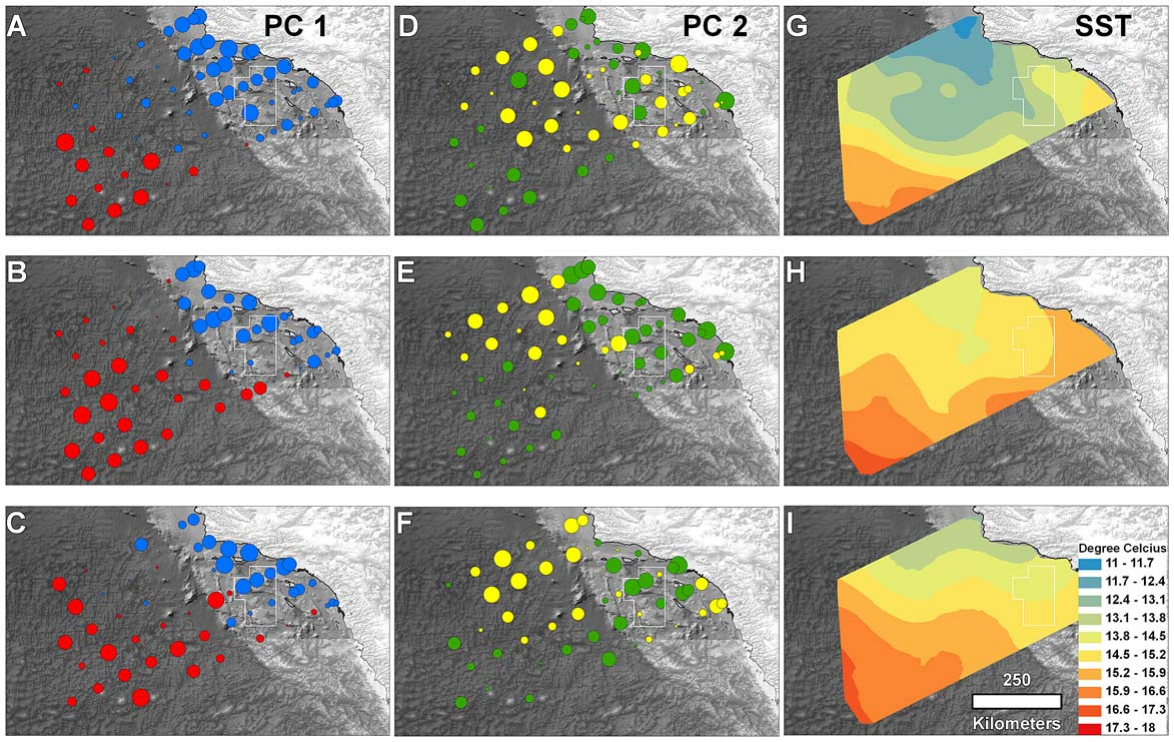
Spatial and temporal variability in assemblage structure and SST at the CalCOFI scale. Principle component 1 (PC1) scores of stations from (A) 2002, (B) 2003 and (C) 2004. Red circles represent positive and blue circles negative loadings on PC1. The size of each circle is proportional to PC absolute value. PC2 scores from (D) 2002, (E) 2003 and (F) 2004. Green circles represent positive and yellow circles negative loadings on PC2. See Table 2 for taxa loadings on each axis. Krig-based images of sea surface temperature from (G) 2002, (H) 2003 and (I) 2004.

Although our results suggest that broad-scale processes affected the CCA assemblage in a way that was not fully apparent by examining local conditions, there is evidence that local processes strongly impacted the CCA assemblage in 2002 as environmental covariates explained more than twice as much variation in this year than in other years. This result might be driven by interannual variability in mesoscale oceanographic structure. The Southern California Bight is bathymetrically heterogeneous and contains several deep basins with steep vertical drops that can induce the formation of local fronts (Fig. 1). Nishimoto and Washburn [43], for example, documented a mesoscale eddy just north of our study area that affected significantly the distribution of late-stage fish larvae in 1998 but found no evidence of the eddy or spatially-structured fish distributions the following year. Working within the CCA region, McClatchie et al. [12] documented a strong north-south front that hugged the ridge south from San Nicholas Island (Fig. 1A) in 2010. This front separated warm, saline water in the east and cool, fresher water in the west, and there were different zooplankton, larval fish, and egg (fish and squid) assemblages on either side of and within the front. In our study there was a clear east-west gradient in both species distributions and SST within the CCA in 2002 but less distinct transitions in other years (Fig. 4, 5). These results suggest that local oceanographic structure contributed more to assemblage structure within the CCA in 2002 than other years and imply that both local and broad-scale oceanographic dynamics can combine to affect the distribution of ichthyoplankton within the CCA.

These results stress the importance of being cognizant of sampling scale when describing species-environment relationships [44]. Although ichthyoplankton studies from around the world documented differences in assemblage structure from very small (e.g., <1-m; [45]) to very large spatial scales (e.g, 4000-km; [6]), explicit evaluations of assemblage-environment relationships across sampling scales within a study are rare. Among the existing multi-scale investigations, Catalan et al. [46] quantified ichthyoplankton assemblage structure from a mesoscale sampling grid (stations separated by 18 km) that was embedded within a macroscale grid (stations separated by 40 km) off the coast of Spain and Portugal. Similar to our results they found consistent differences in assemblage composition between onshelf and offshelf stations at the macroscale. In addition, they discovered through mesoscale sampling that the spatial boundary separating assemblages was porous as taxa with primarily onshelf affinities were advected to offshore areas. Another multi-scale study from the Gulf of Mexico demonstrated that different oceanographic variables best explained ichthyoplankton distribution patterns at fine (1-km), meso (10-km) and coarse (100-km) scale sampling perspectives [47]. These results stress how examining ichthyoplankton from multiple perspectives is important given the inherently dynamic nature of fish distribution.

Ultimately, the aim of future/ongoing research is to compare icthyoplankton assemblage data reported here with future samples to ascertain whether the MPA has an effect on the local production of fishes. Given the findings of this initial effort several recommendations can be made to augment the efficacy of future sampling in this and other ichthyoplankton assemblage studies. First, we found that the CCA assemblage was highly variable at an annual time scale. However, our results are based on only three years of sampling and additional annual sampling is necessary to evaluate how these fluctuations compare to long-term trends. Second, we showed that assemblages were likely affected by unmeasured environmental covariates, endogenous behavior by the species, and/or a mismatch between the scale of sampling and the response of species to the environment. To better resolve the relative importance of these factors future sampling needs to quantify additional oceanographic covariates such as salinity, current velocity and oxygen in concert with species sampling. Third, we obtained insight into processes that affected the CCA assemblage by also sampling at the larger CalCOFI scale. This result emphasizes the importance of recognizing that oceanic MPAs are nested in a broader system where forces external to the reserve can affect assemblage structure within the reserve. Hence, our results stress the need for multi-scale monitoring to elucidate the cause of species fluctuation in MPAs. Our findings set the stage for documentation of the effect of MPAs on fisheries production, which is a critical, yet largely unresolved question in the study of MPAs.

## Supporting Information

Figure S1 **Hellinger-transformed values of oceanic species in the CCA from 2002–2004.** (A–C) *Nanobrachium* ritteri; (D–F) *Protomyctophum crockeri*; (G–I) *Symbolophorous californiensis*. The white border depicts the boundary of the CCA in this and all Supplemental Figures. The order in which taxa are presented is based on their habitat affinities as defined by [31]. The size of a circle is proportional to its value which ranges between 0 and 1.
(TIF)

Figure S2 **Hellinger-transformed values of two oceanic and one coastal-oceanic species in the CCA from 2002–2004.** (A–C) *Diogenicthys atlanticus* (oceanic); (D–F) *Stenobrachius leucopsarus* (oceanic); (G–I) *Leuroglossus stilbius* (coastal-oceanic).
(TIF)

Figure S3 **Hellinger-transformed values of coastal-oceanic species in the CCA from 2002–2004.** (A–C) *Lipolagus ochotensis*; (D–F) *Merluccius productus*; (G–I) *Engraulis mordax*.
(TIF)

Figure S4 **Hellinger-transformed values of benthic taxa in the CCA from 2002–2004.** (A–C) *Sebastes* spp.; (D–F) *Sebastes jordani*; (G–I) *Sebastes paucispinis*.
(TIF)

Figure S5 **Hellinger-transformed values of oceanic species in the CalCOFI domain from 2002–2004.** (A–C) *Nanobrachium* ritteri; (D–F) *Protomyctophum crockeri*; (G–I) *Symbolophorous californiensis*.
(TIF)

Figure S6 **Hellinger-transformed values of two oceanic and one coastal-oceanic species in the CalCOFI domain from 2002–2004.** (A–C) *Diogenicthys atlanticus* (oceanic); (D–F) *Stenobrachius leucopsarus* (oceanic); (G–I) *Leuroglossus stilbius* (coastal-oceanic).
(TIF)

Figure S7 **Hellinger-transformed values of coastal-oceanic species in the CalCOFI domain from 2002–2004.** (A–C) *Lipolagus ochotensis*; (D–F) *Merluccius productus*; (G–I) *Engraulis mordax*.
(TIF)

Figure S8 **Hellinger-transformed values of benthic taxa in the CalCOFI domain from 2002–2004.** (A–C) *Sebastes* spp.; (D–F) *Sebastes jordani*; (G–I) *Sebastes paucispinis*.
(TIF)

Table S1 **Complete list of taxa sampled in the Cowcod Conservation Area and CalCOFI.**
(DOCX)
