# Supplementary figures and images for: Multi-Scale Sampling to Evaluate Assemblage Dynamics in an Oceanic Marine Reserve
Source: PLoS One. 2012 Mar 20;7(3):e33131. doi: 10.1371/journal.pone.0033131 (PMC3308965; doi:10.1371/journal.pone.0033131)

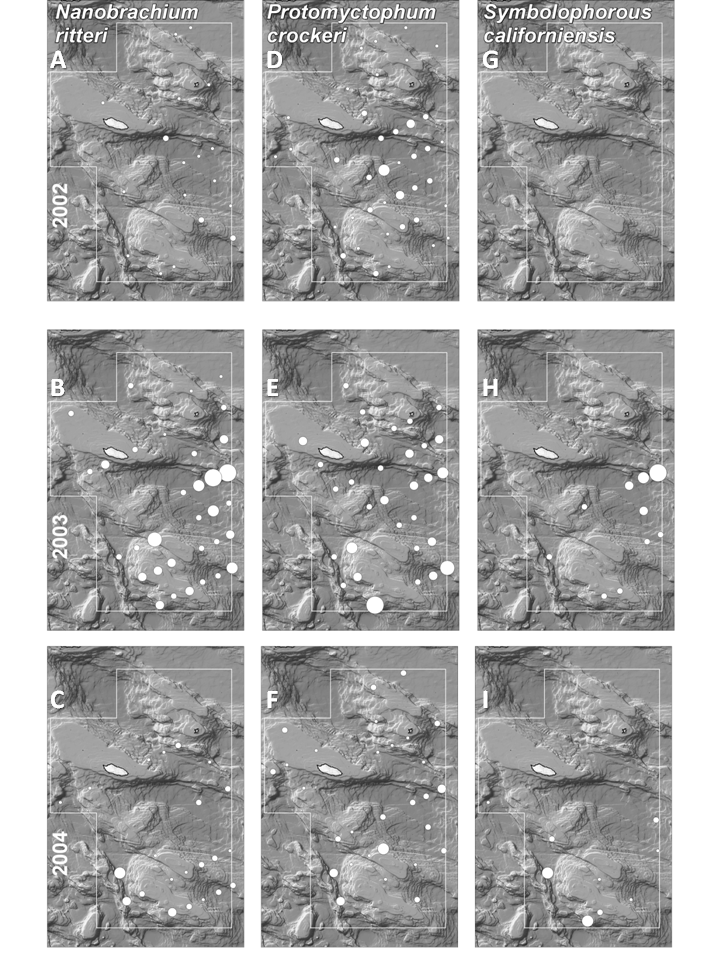

Supplement: Figure S1 — Hellinger-transformed values of oceanic species in the CCA from 2002–2004. (A–C) Nanobrachium ritteri; (D–F) Protomyctophum crockeri; (G–I) Symbolophorous californiensis. The white border depicts the boundary of the CCA in this and all Supplemental Figures. The order in which taxa are presented is based on their habitat affinities as defined by [31]. The size of a circle is proportional to its value which ranges between 0 and 1. (TIF) [file pone.0033131.s001.tif]

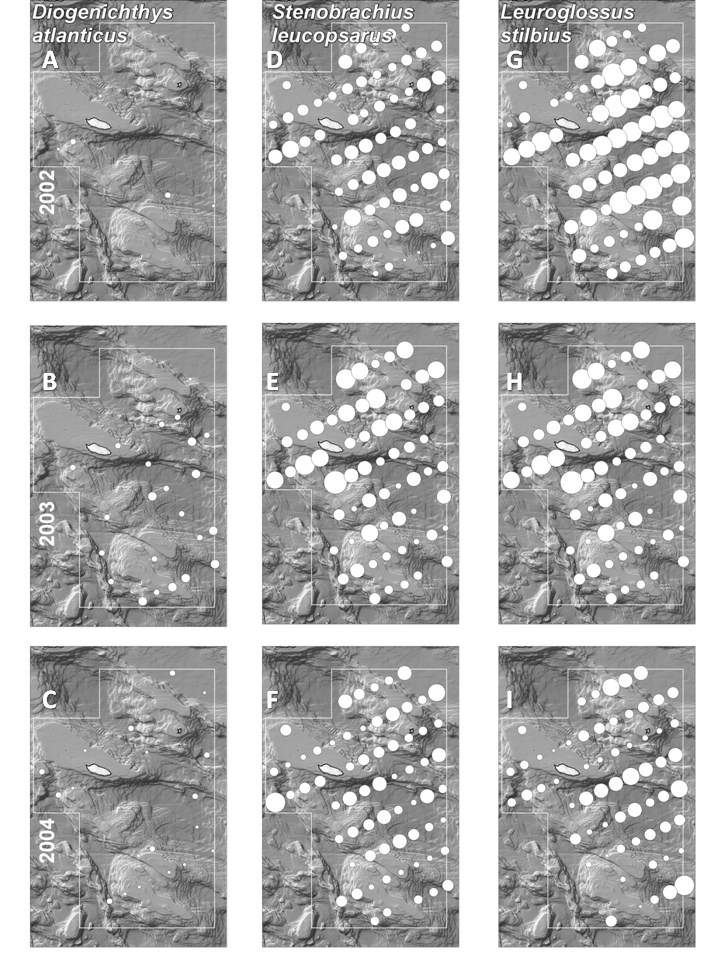

Supplement: Figure S2 — Hellinger-transformed values of two oceanic and one coastal-oceanic species in the CCA from 2002–2004. (A–C) Diogenicthys atlanticus (oceanic); (D–F) Stenobrachius leucopsarus (oceanic); (G–I) Leuroglossus stilbius (coastal-oceanic). (TIF) [file pone.0033131.s002.tif]

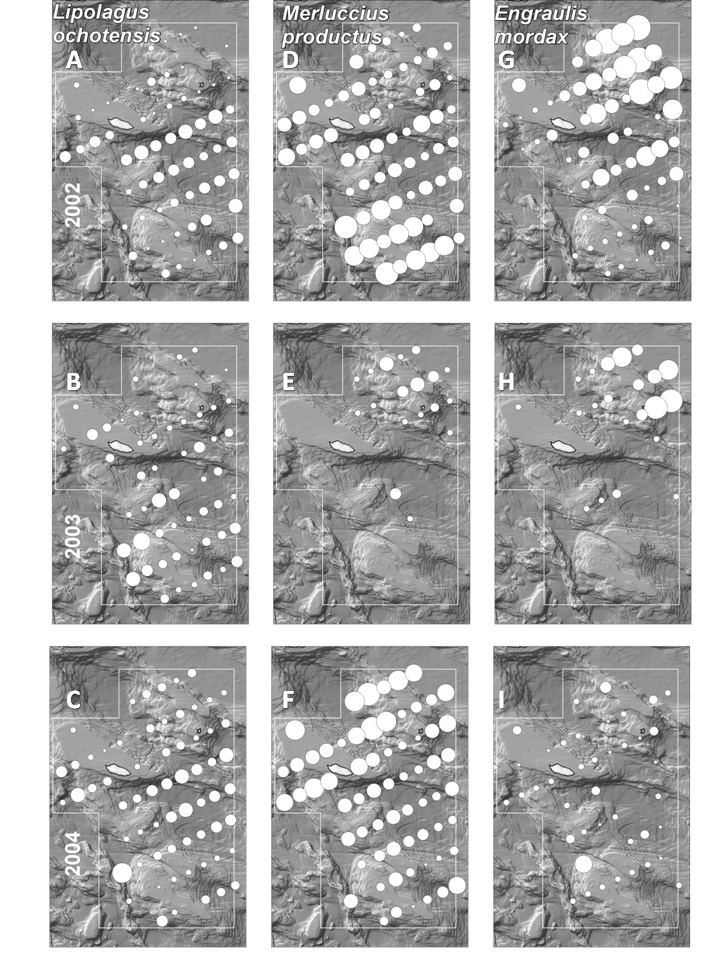

Supplement: Figure S3 — Hellinger-transformed values of coastal-oceanic species in the CCA from 2002–2004. (A–C) Lipolagus ochotensis; (D–F) Merluccius productus; (G–I) Engraulis mordax. (TIF) [file pone.0033131.s003.tif]

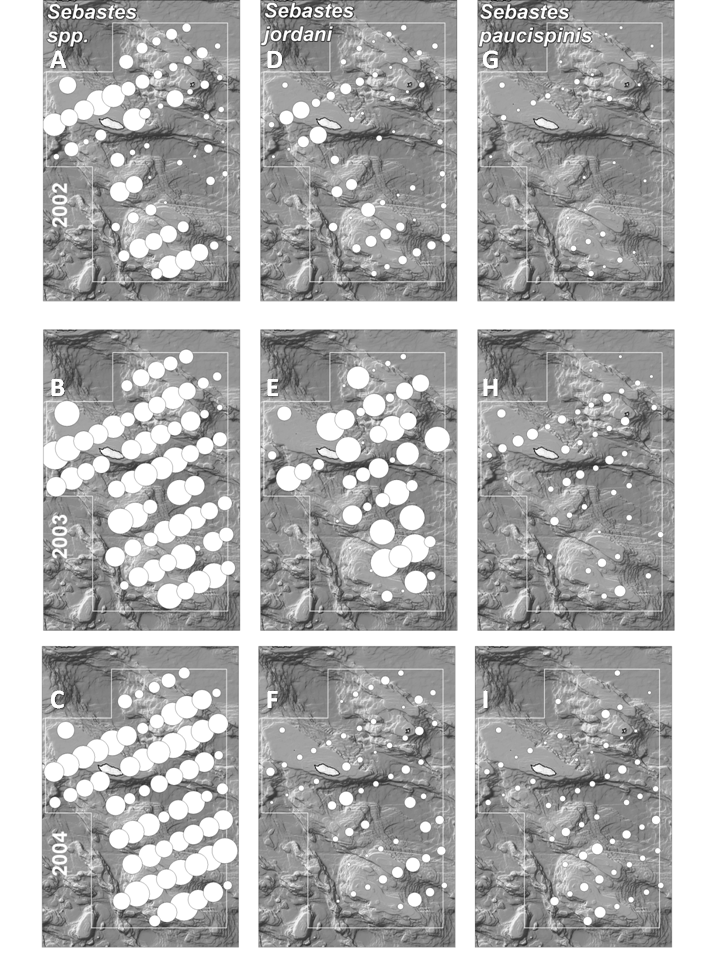

Supplement: Figure S4 — Hellinger-transformed values of benthic taxa in the CCA from 2002–2004. (A–C) Sebastes spp.; (D–F) Sebastes jordani; (G–I) Sebastes paucispinis. (TIF) [file pone.0033131.s004.tif]

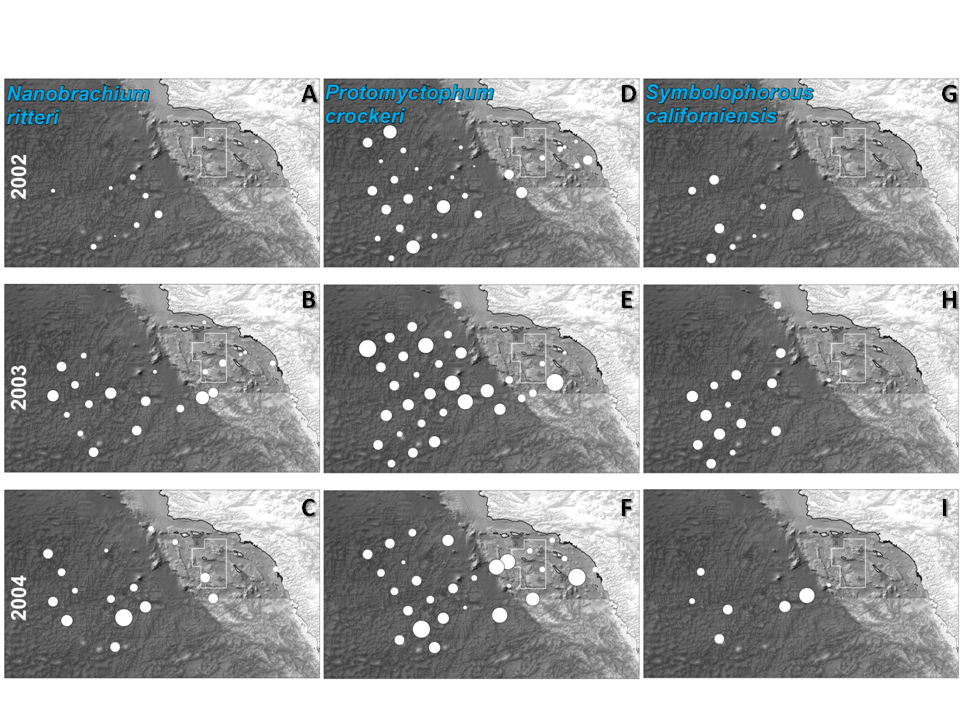

Supplement: Figure S5 — Hellinger-transformed values of oceanic species in the CalCOFI domain from 2002–2004. (A–C) Nanobrachium ritteri; (D–F) Protomyctophum crockeri; (G–I) Symbolophorous californiensis. (TIF) [file pone.0033131.s005.tif]

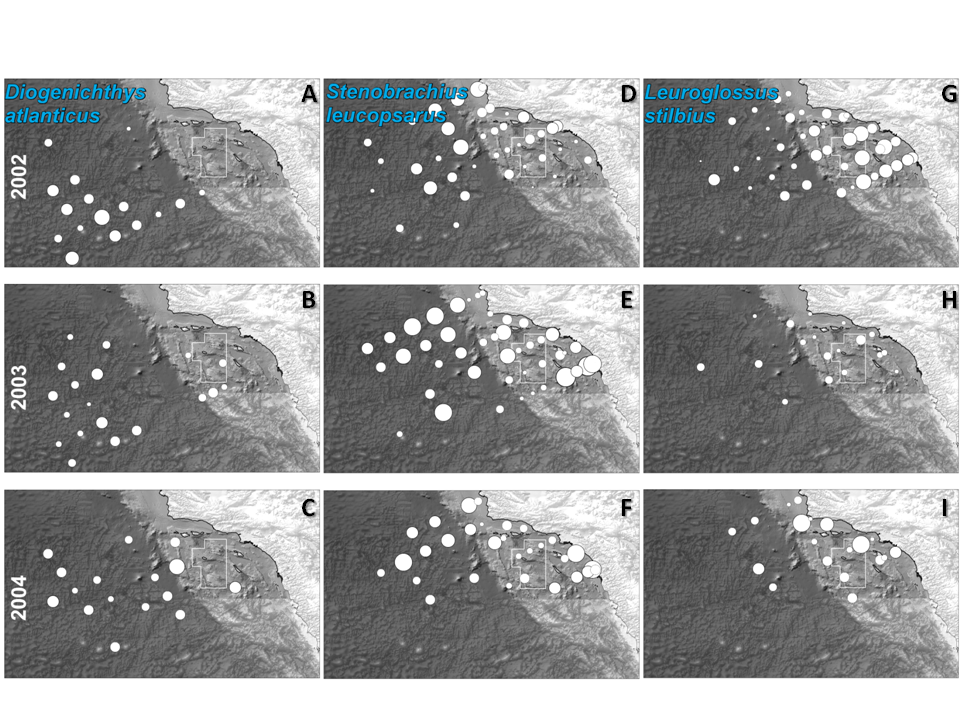

Supplement: Figure S6 — Hellinger-transformed values of two oceanic and one coastal-oceanic species in the CalCOFI domain from 2002–2004. (A–C) Diogenicthys atlanticus (oceanic); (D–F) Stenobrachius leucopsarus (oceanic); (G–I) Leuroglossus stilbius (coastal-oceanic). (TIF) [file pone.0033131.s006.tif]

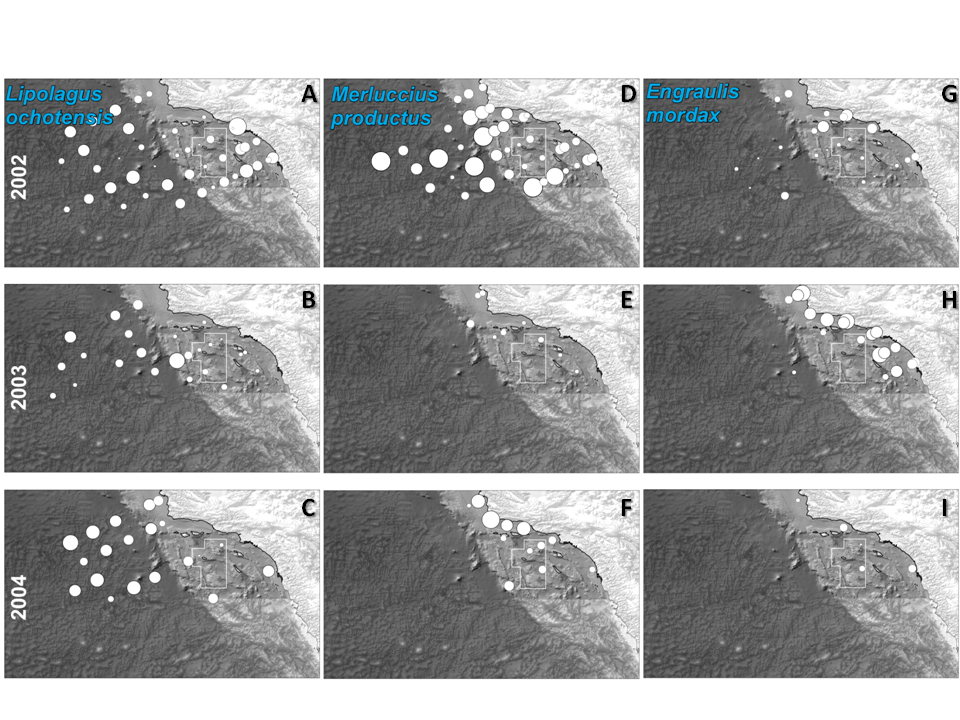

Supplement: Figure S7 — Hellinger-transformed values of coastal-oceanic species in the CalCOFI domain from 2002–2004. (A–C) Lipolagus ochotensis; (D–F) Merluccius productus; (G–I) Engraulis mordax. (TIF) [file pone.0033131.s007.tif]

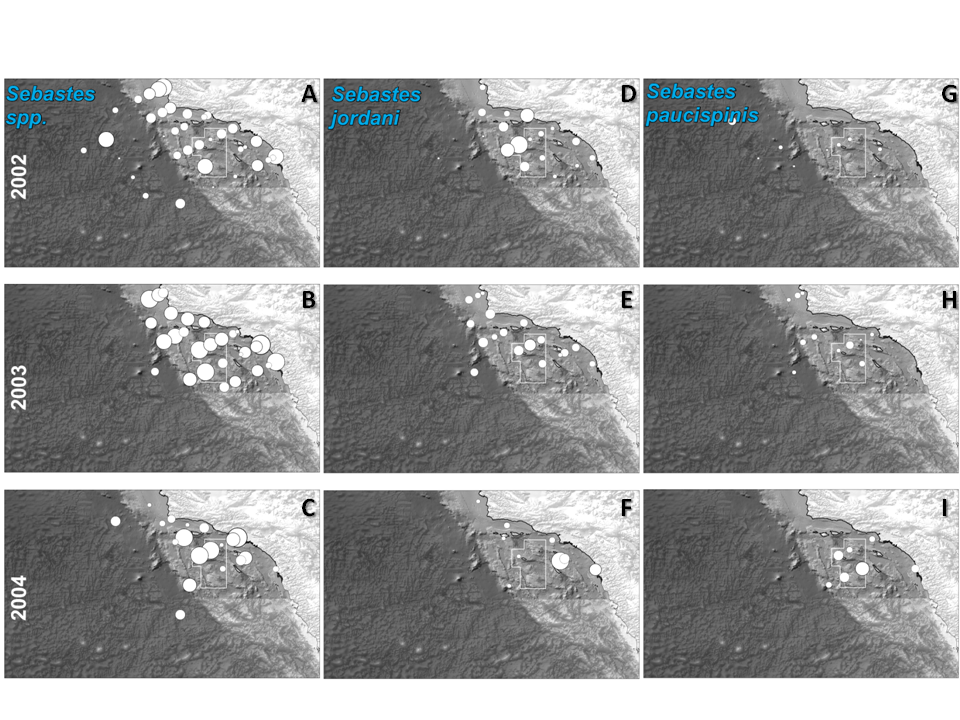

Supplement: Figure S8 — Hellinger-transformed values of benthic taxa in the CalCOFI domain from 2002–2004. (A–C) Sebastes spp.; (D–F) Sebastes jordani; (G–I) Sebastes paucispinis. (TIF) [file pone.0033131.s008.tif]
