# Supplementary material for: Multi-Scale Sampling to Evaluate Assemblage Dynamics in an Oceanic Marine Reserve
Source: PLoS One. 2012 Mar 20;7(3):e33131. doi: 10.1371/journal.pone.0033131 (PMC3308965; doi:10.1371/journal.pone.0033131)
Supplement: Table S1 — Complete list of taxa sampled in the Cowcod Conservation Area and CalCOFI. (DOCX) [file pone.0033131.s009.docx]

**Table S1**

|  |  |  | **Cowcod Conservation Area Domain** | | | | |  |  | **CalCOFI Domain** | | | |  |
| --- | --- | --- | --- | --- | --- | --- | --- | --- | --- | --- | --- | --- | --- | --- |
|  |  |  | **Frequency of Occurrence** | | | **Mean Abundance** | | | **Frequency of Occurrence** | | | **Mean Abundance** | | |
| **Taxon** | | **common name** | **2002** | **2003** | **2004** | **2002** | **2003** | **2004** | **2002** | **2003** | **2004** | **2002** | **2003** | **2004** |
| Cyematidae | |  |  |  |  |  |  |  |  |  |  |  |  |  |
|  | *Cyema atrum* | Bobtail snipe-eel | 0 | 0 | 0 | 0 | 0 | 0 | 0.02 | 0 | 0 | 0.08 | 0 | 0 |
| Clupeidae | |  |  |  |  |  |  |  |  |  |  |  |  |  |
|  | *Sardinops sagax* | Pacific sardine | 0.09 | 0.01 | 0.13 | 32.85 | 0.07 | 0.81 | 0 | 0.03 | 0 | 0 | 0.36 | 0 |
| Engraulidae | |  |  |  |  |  |  |  |  |  |  |  |  |  |
|  | *Engraulis mordax* | Northern anchovy | 0.91 | 0.31 | 0.63 | 302.72 | 24.26 | 15.60 | 0.35 | 0.29 | 0.08 | 12.80 | 18.49 | 0.51 |
| Argentinidae | |  |  |  |  |  |  |  |  |  |  |  |  |  |
|  | *Argentina sialis* | Pacific argentine | 0.20 | 0.01 | 0.09 | 1.04 | 0.08 | 0.51 | 0.03 | 0.02 | 0.05 | 0.22 | 0.07 | 0.29 |
| Bathylagidae | |  |  |  |  |  |  |  |  |  |  |  |  |  |
|  | *Leuroglossus stilbius* | Cal. smoothtongue | 1.00 | 0.79 | 0.91 | 286.04 | 29.01 | 47.84 | 0.63 | 0.28 | 0.30 | 52.74 | 4.29 | 13.80 |
|  | *Bathylagus pacificus* | Pacific blacksmelt | 0.06 | 0.04 | 0.09 | 0.36 | 0.22 | 0.44 | 0.02 | 0 | 0 | 0.07 | 0 | 0 |
|  | *Bathylagoides wesethi* | Snubnose blacksmelt | 0 | 0 | 0 | 0 | 0 | 0 | 0 | 0.06 | 0.05 | 0 | 0.36 | 0.35 |
|  | *Lipolagus ochotensis* | Popeye blacksmelt | 0.92 | 0.81 | 0.94 | 44.32 | 11.81 | 31.37 | 0.66 | 0.37 | 0.31 | 30.85 | 3.24 | 8.59 |
|  | *Pseudobathylagus milleri* | Robust blacksmelt | 0 | 0 | 0 | 0 | 0 | 0 | 0 | 0 | 0.02 | 0 | 0 | 0.07 |
| Microstomatidae | |  |  |  |  |  |  |  |  |  |  |  |  |  |
|  | *Microstoma spp.* | Dusky pencilsmelt | 0.05 | 0 | 0.03 | 0.22 | 0 | 0.14 | 0.06 | 0 | 0.03 | 0.35 | 0 | 0.20 |
| Opisthoproctidae | |  |  |  |  |  |  |  |  |  |  |  |  |  |
|  | *Dolichopteryx longipes* | Brownsnout spookfish | 0 | 0 | 0 | 0 | 0 | 0 | 0.02 | 0 | 0 | 0.14 | 0 | 0 |
| Gonostomatidae | |  |  |  |  |  |  |  |  |  |  |  |  |  |
|  | *Cyclothone acclinidens* | Benttooth bristlemouth | 0 | 0 | 0 | 0 | 0 | 0 | 0 | 0 | 0.02 | 0 | 0 | 0.07 |
|  | *Cyclothone signata* | Showy bristlemouth | 0 | 0.09 | 0.09 | 0 | 0.72 | 0.65 | 0.08 | 0.15 | 0.17 | 0.68 | 1.67 | 1.14 |
| Sternoptychidae | |  |  |  |  |  |  |  |  |  |  |  |  |  |
|  | *Argyropelecus affinis* | Slender hatchetfish | 0.02 | 0 | 0 | 0.07 | 0 | 0 | 0.03 | 0.02 | 0.02 | 0.22 | 0.07 | 0.07 |
|  | *Argyropelecus hemigymnus* | Spurred hatchetfish | 0.02 | 0 | 0.01 | 0.07 | 0 | 0.08 | 0 | 0.03 | 0 | 0 | 0.21 | 0 |
|  | *Argyropelecus lychnus* | Tropical hatchetfish | 0.02 | 0 | 0 | 0.07 | 0 | 0 | 0.03 | 0 | 0.02 | 0.15 | 0 | 0.07 |
|  | *Argyropelecus sladeni* | Lowcrest hatchetfish | 0.18 | 0.04 | 0.04 | 1.02 | 0.22 | 0.21 | 0.12 | 0.08 | 0.06 | 0.98 | 0.42 | 0.44 |
|  | *Danaphos oculatus* | Bottlelight | 0.09 | 0.07 | 0.13 | 0.45 | 0.46 | 0.73 | 0.12 | 0.09 | 0.11 | 1.24 | 0.42 | 0.77 |
|  | *Sternoptyx spp.* | Dollar hatchetfish | 0.02 | 0.01 | 0 | 0.07 | 0.08 | 0 | 0.03 | 0 | 0 | 0.25 | 0 | 0 |
| Phosichthyidae | |  |  |  |  |  |  |  |  |  |  |  |  |  |
|  | *Ichthyococcus irregularis* | Bulldog lightfish | 0 | 0.01 | 0.01 | 0 | 0.08 | 0.07 | 0.03 | 0 | 0 | 0.22 | 0 | 0 |
|  | *Vinciguerria lucetia* | Panama lightfish | 0.02 | 0.01 | 0.07 | 0.07 | 0.07 | 0.71 | 0.18 | 0.18 | 0.08 | 4.61 | 3.27 | 0.42 |
| Chauliodontidae | |  |  |  |  |  |  |  |  |  |  |  |  |  |
|  | *Chauliodus macouni* | Pacific viperfish | 0.02 | 0.07 | 0.06 | 0.08 | 0.37 | 0.28 | 0.03 | 0.03 | 0.05 | 0.14 | 0.14 | 0.29 |
| Stomiidae | |  |  |  |  |  |  |  |  |  |  |  |  |  |
|  | *Stomias atriventer* | Blackbelly dragonfish | 0.08 | 0.04 | 0.10 | 0.59 | 0.24 | 0.49 | 0.02 | 0.06 | 0.02 | 0.16 | 0.28 | 0.07 |
| Malacosteidae | |  |  |  |  |  |  |  |  |  |  |  |  |  |
|  | *Aristostomias scintillans* | Shining loosejaw | 0 | 0 | 0 | 0 | 0 | 0 | 0.02 | 0.02 | 0 | 0.08 | 0.07 | 0 |
| Idiacanthidae | |  |  |  |  |  |  |  |  |  |  |  |  |  |
|  | *Idiacanthus antrostomus* | Pacific blackdragon | 0 | 0.01 | 0 | 0 | 0.08 | 0 | 0.12 | 0.05 | 0.06 | 0.76 | 0.34 | 0.28 |
| Scopelarchidae | |  |  |  |  |  |  |  |  |  |  |  |  |  |
|  | *Benthalbella dentata* | Northern pearleye | 0.03 | 0.07 | 0.03 | 0.14 | 0.38 | 0.15 | 0 | 0.03 | 0.05 | 0 | 0.22 | 0.28 |
|  | *Rosenblattichthys volucris* | Chubby pearleye | 0 | 0 | 0 | 0 | 0 | 0 | 0.02 | 0.02 | 0 | 0.08 | 0.07 | 0 |
|  | *Scopelarchus analis* | Blackbelly pearleye | 0 | 0 | 0 | 0 | 0 | 0 | 0.02 | 0.03 | 0 | 0.08 | 0.14 | 0 |
| Notosudidae | |  |  |  |  |  |  |  |  |  |  |  |  |  |
|  | *Scopelosaurus spp.* | Paperbone | 0 | 0 | 0 | 0 | 0 | 0 | 0.02 | 0.02 | 0.02 | 0.15 | 0.07 | 0.07 |
| Paralepididae | |  |  |  |  |  |  |  |  |  |  |  |  |  |
|  | *Arctozenus risso* | Ribbon barracudina | 0 | 0 | 0 | 0 | 0 | 0 | 0 | 0 | 0.02 | 0 | 0 | 0.15 |
|  | *Lestidiops ringens* | Slender barracudina | 0.02 | 0.16 | 0.12 | 0.07 | 0.95 | 0.56 | 0.05 | 0.14 | 0.03 | 0.29 | 0.68 | 0.20 |
| Myctophidae | |  |  |  |  |  |  |  |  |  |  |  |  |  |
|  | *Ceratoscopelus townsendi* | Dogtooth lampfish | 0 | 0 | 0 | 0 | 0 | 0 | 0.11 | 0.09 | 0.02 | 1.43 | 0.49 | 0.07 |
|  | *Diaphus spp.* | Headlightfish | 0.02 | 0 | 0 | 0.08 | 0 | 0 | 0.03 | 0.02 | 0 | 0.15 | 0.07 | 0 |
|  | *Nannobrachium bristori* | lampfish | 0 | 0 | 0 | 0 | 0 | 0 | 0.02 | 0.02 | 0 | 0.08 | 0.07 | 0 |
|  | *Nannobrachium regale* | Pinpoint lampfish | 0.02 | 0.01 | 0 | 0.07 | 0.07 | 0 | 0 | 0 | 0 | 0 | 0 | 0 |
|  | *Nannobrachium ritteri* | Broadfin lampfish | 0.27 | 0.49 | 0.33 | 2.04 | 4.76 | 3.36 | 0.15 | 0.34 | 0.27 | 1.53 | 4.23 | 2.17 |
|  | *Notolychnus valdiviae* | Topside lampfish | 0 | 0 | 0 | 0 | 0 | 0 | 0.02 | 0 | 0 | 0.08 | 0 | 0 |
|  | *Stenobrachius leucopsarus* | Northern lampfish | 0.98 | 0.93 | 0.99 | 148.66 | 53.10 | 69.53 | 0.62 | 0.66 | 0.50 | 44.45 | 24.50 | 18.76 |
|  | *Triphoturus mexicanus* | Mexican lampfish | 0 | 0.01 | 0.01 | 0 | 0.22 | 0.06 | 0 | 0.05 | 0 | 0 | 0.35 | 0 |
|  | *Diogenichthys atlanticus* | Longfin lanternfish | 0.08 | 0.34 | 0.28 | 0.45 | 2.29 | 1.55 | 0.25 | 0.29 | 0.25 | 5.76 | 3.27 | 2.26 |
|  | *Electrona risso* | Chubby flashlightfish | 0 | 0.01 | 0 | 0 | 0.08 | 0 | 0.02 | 0 | 0 | 0.07 | 0 | 0 |
|  | *Hygophum reinhardtii* | Slender lanternfish | 0 | 0 | 0 | 0 | 0 | 0 | 0.03 | 0 | 0.02 | 0.22 | 0 | 0.07 |
|  | *Myctophum nitidulum* | Pearly lanternfish | 0 | 0 | 0 | 0 | 0 | 0 | 0.05 | 0 | 0 | 0.53 | 0 | 0 |
|  | *Protomyctophum crockeri* | California flashlightfish | 0.59 | 0.46 | 0.43 | 7.59 | 4.47 | 3.81 | 0.48 | 0.49 | 0.45 | 7.33 | 6.72 | 4.23 |
|  | *Symbolophorus californiensis* | California lanternfish | 0 | 0.15 | 0.13 | 0 | 1.13 | 0.88 | 0.12 | 0.25 | 0.13 | 1.54 | 3.20 | 1.29 |
|  | *Symbolophorus evermanni* | Evermann's lanternfish | 0 | 0.01 | 0 | 0 | 0.55 | 0 | 0 | 0 | 0 | 0 | 0 | 0 |
|  | *Tarletonbeania crenularis* | Blue lanternfish | 0.42 | 0.27 | 0.04 | 3.49 | 2.19 | 0.20 | 0.20 | 0.15 | 0.08 | 2.49 | 1.01 | 0.56 |
| Macrouridae | |  |  |  |  |  |  |  |  |  |  |  |  |  |
|  | *Coryphaenoides pectoralis* | Giant grenadier | 0.02 | 0 | 0 | 0.07 | 0 | 0 | 0 | 0 | 0 | 0 | 0 | 0 |
| Merlucciidae | |  |  |  |  |  |  |  |  |  |  |  |  |  |
|  | *Merluccius productus* | Pacific hake | 1.00 | 0.27 | 0.97 | 252.80 | 8.79 | 117.29 | 0.62 | 0.14 | 0.19 | 338.59 | 1.42 | 9.23 |
| Ophidiidae | |  |  |  |  |  |  |  |  |  |  |  |  |  |
|  | *Chilara taylori* | Spotted cusk-eel | 0.02 | 0 | 0 | 0.08 | 0 | 0 | 0 | 0 | 0 | 0 | 0 | 0 |
| Bythitidae | |  |  |  |  |  |  |  |  |  |  |  |  |  |
|  | *Brosmophycis marginata* | Red brotula | 0.03 | 0 | 0 | 0.14 | 0 | 0 | 0 | 0 | 0 | 0 | 0 | 0 |
| Gobiesocidae | |  |  |  |  |  |  |  |  |  |  |  |  |  |
|  |  | unidentified clingfish | 0 | 0 | 0.01 | 0 | 0 | 0.07 | 0 | 0 | 0 | 0 | 0 | 0 |
| Atherinidae | |  |  |  |  |  |  |  |  |  |  |  |  |  |
|  | *Atherinopsis californiensis* | Jacksmelt | 0 | 0 | 0 | 0 | 0 | 0 | 0.02 | 0 | 0 | 0.07 | 0 | 0 |
| Melamphaidae | |  |  |  |  |  |  |  |  |  |  |  |  |  |
|  | *Melamphaes lugubris* | Highsnout bigscale | 0 | 0 | 0.04 | 0 | 0 | 0.22 | 0.02 | 0.05 | 0.05 | 0.08 | 0.35 | 0.27 |
|  | *Melamphaes parvus* | Little bigscale | 0 | 0.06 | 0.03 | 0 | 0.38 | 0.15 | 0.03 | 0.03 | 0.03 | 0.21 | 0.14 | 0.36 |
|  | *Poromitra crassiceps* | Crested bigscale | 0 | 0 | 0 | 0 | 0 | 0 | 0.02 | 0 | 0 | 0.07 | 0 | 0 |
|  | *Scopelogadus mizolepis bispinosus* | Twospine bigscale | 0 | 0 | 0 | 0 | 0 | 0 | 0 | 0 | 0.02 | 0 | 0 | 0.07 |
| Scorpaenidae | |  |  |  |  |  |  |  |  |  |  |  |  |  |
|  | *Sebastes aurora* | Aurora rockfish | 0.15 | 0.06 | 0.10 | 0.94 | 0.62 | 0.73 | 0.02 | 0.02 | 0 | 0.30 | 0.07 | 0 |
|  | *Sebastes diploproa* | Splitnose rockfish | 0 | 0 | 0 | 0 | 0 | 0 | 0 | 0 | 0.06 | 0 | 0 | 2.45 |
|  | *Sebastes goodei* | Chillipepper rockfish | 0.03 | 0.09 | 0.19 | 0.23 | 0.61 | 1.66 | 0.02 | 0.03 | 0 | 0.16 | 0.13 | 0 |
|  | *Sebastes jordani* | Shortbelly rockfish | 0.82 | 0.69 | 0.87 | 56.48 | 39.22 | 27.95 | 0.28 | 0.28 | 0.14 | 38.35 | 8.36 | 3.04 |
|  | *Sebastes levis* | Cowcod rockfish | 0.11 | 0.15 | 0.24 | 0.88 | 0.79 | 2.19 | 0 | 0.06 | 0 | 0 | 0.29 | 0 |
|  | *Sebastes paucispinis* | Bocaccio rockfish | 0.44 | 0.64 | 0.84 | 6.91 | 8.37 | 15.51 | 0.14 | 0.17 | 0.11 | 1.74 | 1.81 | 7.08 |
|  | *Sebastes spp.* | unidentified rockfish | 0.88 | 0.94 | 0.99 | 250.07 | 135.34 | 345.92 | 0.55 | 0.43 | 0.30 | 43.59 | 35.59 | 32.00 |
|  | *Sebastolobus spp.* | unidentified thornyhead | 0 | 0 | 0.06 | 0 | 0 | 0.35 | 0 | 0 | 0 | 0 | 0 | 0 |
| Hexagrammidae | |  |  |  |  |  |  |  |  |  |  |  |  |  |
|  | *Hexagrammos decagrammus* | Kelp greenling | 0 | 0 | 0 | 0 | 0 | 0 | 0.02 | 0 | 0 | 0.30 | 0 | 0 |
|  | *Ophiodon elongatus* | Lingcod | 0.02 | 0 | 0.03 | 0.06 | 0 | 0.14 | 0 | 0.02 | 0.02 | 0 | 0.06 | 0.15 |
|  | *Oxylebius pictus* | Painted greenling | 0.08 | 0.04 | 0.04 | 0.36 | 0.22 | 0.22 | 0 | 0 | 0 | 0 | 0 | 0 |
|  | *Zaniolepis frenata* | Shortspine combfish | 0.02 | 0 | 0.01 | 0.07 | 0 | 0.06 | 0 | 0 | 0 | 0 | 0 | 0 |
|  | *Zaniolepis latipinnis* | Longspine combfish | 0.02 | 0.03 | 0.13 | 0.07 | 0.15 | 0.84 | 0.02 | 0.02 | 0 | 0.15 | 0.07 | 0 |
| Cottidae | |  |  |  |  |  |  |  |  |  |  |  |  |  |
|  | *Scorpaenichthys marmoratus* | Cabezon | 0.03 | 0.01 | 0.03 | 0.14 | 0.08 | 0.21 | 0 | 0.02 | 0 | 0 | 0.06 | 0 |
|  | *Artedius fenestralis* | Padded sculpin | 0 | 0.01 | 0 | 0 | 0.31 | 0 | 0 | 0 | 0 | 0 | 0 | 0 |
|  | *Artedius lateralis* | Smoothhead sculpin | 0 | 0 | 0.04 | 0 | 0 | 0.32 | 0 | 0 | 0 | 0 | 0 | 0 |
|  | *Ascelichthys rhodorus* | Rosylip sculpin | 0.02 | 0 | 0 | 0.15 | 0 | 0 | 0 | 0 | 0 | 0 | 0 | 0 |
|  | *Chitonotus pugetensis* | Roughback sculpin | 0.02 | 0.01 | 0 | 0.20 | 0.07 | 0 | 0 | 0 | 0 | 0 | 0 | 0 |
|  | *Icelinus quadriseriatus* | Yellowchin sculpin | 0.05 | 0.06 | 0.04 | 0.33 | 0.75 | 0.32 | 0 | 0 | 0.02 | 0 | 0 | 0.19 |
|  | *Icelinus spp.* | unidentified sculpin | 0.02 | 0 | 0.03 | 0.08 | 0 | 0.43 | 0 | 0 | 0 | 0 | 0 | 0 |
|  | *Paricelinus hopliticus* | Thornback sculpin | 0.02 | 0 | 0.03 | 0.07 | 0 | 0.22 | 0 | 0 | 0 | 0 | 0 | 0 |
|  | *Radulinus asprellus* | Slim sculpin | 0.05 | 0 | 0 | 0.20 | 0 | 0 | 0 | 0 | 0 | 0 | 0 | 0 |
|  | *Ruscarius creaseri* | Roughcheek sculpin | 0.05 | 0.01 | 0.04 | 0.33 | 0.31 | 0.84 | 0 | 0 | 0.02 | 0 | 0 | 0.07 |
|  | *Ruscarius meanyi* | Puget Sound sculpin | 0 | 0 | 0.03 | 0 | 0 | 0.13 | 0 | 0 | 0 | 0 | 0 | 0 |
|  |  | unidentified sculpin | 0 | 0.01 | 0.01 | 0 | 0.07 | 0.07 | 0 | 0 | 0 | 0 | 0 | 0 |
| Agonidae | |  |  |  |  |  |  |  |  |  |  |  |  |  |
|  | *Bathyagonus pentacanthus* | Bigeye starsnout poacher | 0 | 0.03 | 0.07 | 0 | 0.14 | 0.35 | 0 | 0 | 0 | 0 | 0 | 0 |
|  | *Odontopyxis trispinosa* | Pygmy poacher | 0 | 0 | 0.01 | 0 | 0 | 0.06 | 0 | 0 | 0 | 0 | 0 | 0 |
|  | *Xeneretmus latifrons* | Blacktip poacher | 0 | 0 | 0 | 0 | 0 | 0 | 0 | 0.02 | 0 | 0 | 0.07 | 0 |
|  | *Xeneretmus leiops* | Smootheye poacher | 0.06 | 0.01 | 0.07 | 0.52 | 0.07 | 0.53 | 0 | 0 | 0 | 0 | 0 | 0 |
| Crangidae | |  |  |  |  |  |  |  |  |  |  |  |  |  |
|  | *Trachurus symmetricus* | Jack mackerel | 0 | 0.15 | 0.03 | 0 | 1.84 | 0.23 | 0.02 | 0.02 | 0 | 0.07 | 0.36 | 0 |
| Sciaenidae | |  |  |  |  |  |  |  |  |  |  |  |  |  |
|  | *Genyonemus lineatus* | White croaker | 0.02 | 0 | 0 | 0.08 | 0 | 0 | 0 | 0.05 | 0.02 | 0 | 1.97 | 0.15 |
| Bathymasteridae | |  |  |  |  |  |  |  |  |  |  |  |  |  |
|  | *Rathbunella spp.* | Ronquil | 0 | 0.01 | 0.04 | 0 | 0.06 | 1.48 | 0 | 0.02 | 0.02 | 0 | 0.06 | 0.14 |
| Stichaeidiae | |  |  |  |  |  |  |  |  |  |  |  |  |  |
|  | *Plectobranchus evides* | Bluebarred prickleback | 0 | 0.01 | 0.03 | 0 | 0.22 | 0.16 | 0 | 0 | 0 | 0 | 0 | 0 |
|  |  | unidentified prickleback | 0 | 0.07 | 0 | 0 | 0.40 | 0 | 0.02 | 0 | 0 | 0.06 | 0 | 0 |
| Chiasmodontidae | |  |  |  |  |  |  |  |  |  |  |  |  |  |
|  | *Chiasmodon niger* | Black swallower | 0 | 0 | 0 | 0 | 0 | 0 | 0 | 0.06 | 0 | 0 | 0.34 | 0 |
|  | *Chiasmodon spp.* | unidentified swallower | 0 | 0 | 0.01 | 0 | 0 | 0.07 | 0 | 0 | 0 | 0 | 0 | 0 |
| Labrisomidae | |  |  |  |  |  |  |  |  |  |  |  |  |  |
|  | *Cryptotrema corallinum* | Deepwater kelpfish | 0.02 | 0.07 | 0.01 | 0.07 | 1.23 | 0.47 | 0.03 | 0 | 0 | 1.67 | 0 | 0 |
| Icosteidae | |  |  |  |  |  |  |  |  |  |  |  |  |  |
|  | *Icosteus aenigmaticus* | Ragfish | 0 | 0.01 | 0.01 | 0 | 0.08 | 0.08 | 0.03 | 0 | 0 | 0.23 | 0 | 0 |
| Gobiidae | |  |  |  |  |  |  |  |  |  |  |  |  |  |
|  | *Ilypnus gilberti* | Cheekspot goby | 0 | 0 | 0 | 0 | 0 | 0 | 0 | 0.02 | 0 | 0 | 0.06 | 0 |
|  | *Lepidogobius lepidus* | Bay goby | 0 | 0 | 0 | 0 | 0 | 0 | 0 | 0.02 | 0 | 0 | 0.38 | 0 |
|  | *Rhinogobiops nicholsii* | Blackeye goby | 0.30 | 0.15 | 0.51 | 2.14 | 1.22 | 3.49 | 0.02 | 0.06 | 0.09 | 0.15 | 0.28 | 0.64 |
| Centrolophidae | |  |  |  |  |  |  |  |  |  |  |  |  |  |
|  | *Icichthys lockingtoni* | Medusafish | 0.03 | 0 | 0.03 | 0.35 | 0 | 0.23 | 0.12 | 0.02 | 0 | 1.25 | 0.07 | 0 |
| Tetragonuridae | |  |  |  |  |  |  |  |  |  |  |  |  |  |
|  | *Tetragonurus cuvieri* | Smalleye squaretail | 0 | 0 | 0 | 0 | 0 | 0 | 0 | 0.03 | 0 | 0 | 0.14 | 0 |
| Paralichthyidae | |  |  |  |  |  |  |  |  |  |  |  |  |  |
|  | *Citharichthys sordidus* | Pacific sanddab | 0.44 | 0.04 | 0.66 | 6.94 | 0.23 | 9.81 | 0.34 | 0.09 | 0.20 | 4.72 | 0.68 | 1.94 |
|  | *Citharichthys stigmaeus* | Speckled sanddab | 0.42 | 0.15 | 0.43 | 3.85 | 0.75 | 2.93 | 0.26 | 0.06 | 0.08 | 5.46 | 0.58 | 0.73 |
|  | *Paralichthys californicus* | California halibut | 0 | 0 | 0 | 0 | 0 | 0 | 0 | 0 | 0 | 0 | 0 | 0 |
| Pleuronectidae | |  |  |  |  |  |  |  |  |  |  |  |  |  |
|  | *Lepidopsetta bilineata* | Rock sole | 0.02 | 0 | 0 | 0.13 | 0 | 0 | 0 | 0 | 0 | 0 | 0 | 0 |
|  | *Lyopsetta exilis* | Slender sole | 0.20 | 0.01 | 0.04 | 1.30 | 0.07 | 0.36 | 0 | 0 | 0 | 0 | 0 | 0 |
|  | *Microstomus pacificus* | Dover sole | 0 | 0.01 | 0.01 | 0 | 0.08 | 0.07 | 0 | 0 | 0 | 0 | 0 | 0 |
|  | *Parophrys vetulus* | English sole | 0.02 | 0 | 0.07 | 0.08 | 0 | 0.35 | 0.02 | 0 | 0.02 | 0.06 | 0 | 0.07 |
|  | *Pleuronichthys coenosus* | C-O turbot | 0.02 | 0 | 0.04 | 0.06 | 0 | 0.20 | 0 | 0 | 0 | 0 | 0 | 0 |
|  | *Pleuronichthys decurrens* | Curlfin turbot | 0 | 0 | 0.03 | 0 | 0 | 0.15 | 0 | 0 | 0 | 0 | 0 | 0 |
|  | *Pleuronichthys verticalis* | Hornyhead turbot | 0 | 0 | 0 | 0 | 0 | 0 | 0 | 0.02 | 0.03 | 0 | 0.06 | 0.14 |
